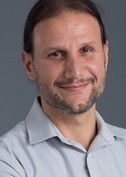# Urine in Bioelectrochemical Systems: An Overall Review

**DOI:** 10.1002/celc.201901995

**Published:** 2020-03-06

**Authors:** Carlo Santoro, Maria Jose Salar Garcia, Xavier Alexis Walter, Jiseon You, Pavlina Theodosiou, Iwona Gajda, Oluwatosin Obata, Jonathan Winfield, John Greenman, Ioannis Ieropoulos

**Affiliations:** ^1^ Bristol BioEnergy Centre Bristol Robotics Laboratory, T-Block, UWE Coldharbour Lane Bristol BS16 1QY UK; ^2^ Biological, Biomedical and Analytical Sciences, UWE Coldharbour Lane Bristol BS16 1QY UK

**Keywords:** urine, microbial fuel cell, bioelectrochemical systems, nitrogen, phosphorous

## Abstract

In recent years, human urine has been successfully used as an electrolyte and organic substrate in bioelectrochemical systems (BESs) mainly due of its unique properties. Urine contains organic compounds that can be utilised as a fuel for energy recovery in microbial fuel cells (MFCs) and it has high nutrient concentrations including nitrogen and phosphorous that can be concentrated and recovered in microbial electrosynthesis cells and microbial concentration cells. Moreover, human urine has high solution conductivity, which reduces the ohmic losses of these systems, improving BES output. This review describes the most recent advances in BESs utilising urine. Properties of neat human urine used in state‐of‐the‐art MFCs are described from basic to pilot‐scale and real implementation. Utilisation of urine in other bioelectrochemical systems for nutrient recovery is also discussed including proofs of concept to scale up systems.

## Graphical abstract

**The power of urine**: Urine has unique characteristics that allow its extensive utilization in bioelectrochemical systems such as microbial fuel cell, microbial electrolysis cell, and bioelectroconcentration cell for electricity production, wastewater treatment, and nutrients recovery.

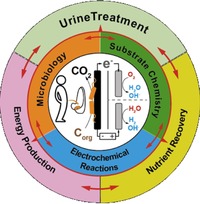

## 1 Introduction

Luigi Galvani conducted the first electrochemical and bioelectrochemical experiment in 1780^1^ where he demonstrated that the muscles of dead frog legs move when struck by an electric spark. Michael Cresse Potter, an English botanist, reported that microorganisms were capable of electron transfer, using synthetic mediators inside a system where redox potential was facilitated using a salt bridge.^2^ This is widely recognised as the first bioelectrochemical system (BES) experiment ever performed, particularly using a microbial fuel cell (MFC). Several advances have since been reported over the years^3^ but the breakthrough discovery came in the 90s when it was demonstrated that bacteria could transfer electrons to anode electrodes without the support of external redox mediators or catabolites.^4^, ^5^

In the past 20 years, there has been a plethora of organic compounds used as substrates/fuels for MFCs, resulting in useful electricity being generated.^6^, ^7^ These substances varied from single and simple organic molecules to complex organic compounds. Industrial wastewater containing diverse simple and complex organic pollutants have been successfully employed as fuel in MFCs.^6^, ^7^ The anodic biofilm in itself is complex and consists of a mixture of electroactive and fermentative microorganisms that coexist synergistically.^8^, ^9^ Electroactive microorganisms are capable of transforming simple organic molecules into electricity.^10^ Therefore, a synergistic interaction between electroactive and fermentative organisms is expected with the latter being responsible for breaking down complex molecules into substrates, which the electroactive bacteria can easily degrade.^8^, ^9^

Human urine is an interesting substrate containing a wide range of organics and nutrients.^11^ Human urine has generated considerable interest in the field of wastewater treatment especially in the past 20 years where source‐separation has been considered. In fact, it has been calculated that human urine is responsible for 10 % of the total chemical oxygen demand (COD), 75 % of total nitrogen and 50 % of phosphorous in municipal wastewater.^12^ Therefore, if urine was treated separately, a recovery of nutrients (N and P) will be more efficient coming from a more concentrated wastewater.^13^, ^14^, ^15^

The use of human urine as substrate in bioelectrochemical systems was firstly introduced almost simultaneously by scientists from the University of the West of England (UK)^16^ Wetsus and Wageningen University (The Netherlands)^17^ and University of Science & Technology of China (China)^18^ with manuscripts published in 2012. The first paper submitted and published was from Ieropoulos *et al*. and the work focused on producing electricity using a microbial fuel cell fed with human urine.^16^ Kuntke *et al*. focused on recovering ammonia at the cathode while still producing useful electricity^17^ whereas Zang *et al*. used urine as fuel for MFCs with the intention of optimising phosphorous recovery.^18^

Human urine contains valuable organic molecules that can be used as fuel in MFCs as well as a high concentration of ammonium ions and ammonia and phosphates that can be valorised in microbial electrolysis cells (MEC)^19^ or bioelectrochemical concentration cells (BEC).^20^ Another important characteristic of urine is the high solution conductivity (>20 mS cm^−1^), which reduces internal losses in electrochemical systems. In fact, high solution conductivity is responsible for decreasing the electrolyte ohmic resistance and therefore enhancing the electrochemical output.^21^

**Figure 1:**
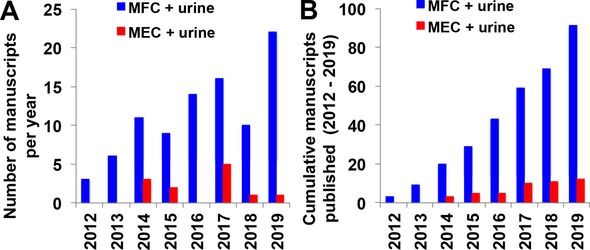
Quantitative analysis of the scientific literature on microbial fuel cells and microbial electrolysis cell (Source: Scopus, November 2019). The search was conducted by inserting the words “microbial fuel cell AND urine” or “microbial electrolysis cell AND urine” that could be found in the title or keywords or abstract.

Since the first reports of using urine in bioelectrochemical systems, there have been a number of experiments conducted over the last 8–9 years. The increasing number of manuscripts published demonstrates an expanding research field. A quantitative search was conducted using Scopus Scientific database; Figure 1.A highlights the manuscripts published annually and Figure 1.B shows the cumulative manuscripts published until now for MFC and MEC fed with urine.

Recently, four critical reviews have been published, focusing on the possibility of utilising bioelectrochemical systems for: i) remediation of nitrate and perchlorate contaminated water;^22^ ii) recovery of nitrogen, phosphorous, water and gaseous valuable products from wastewater;^23^ iii) nitrogen removal and recovery in wastewater treatment systems;^24^ iv) nutrients recovery from urine.^25^

The current review attempts to collate all the main advances reported on the use of neat urine in BESs in the last few years for the purpose of generating electricity and recovering nutrients, which is not covered by existing publications. Firstly, the properties of human urine are discussed in detail. Secondly, the utilisation of urine in MFCs along with the most recent MFC approaches is highlighted. Thirdly, natural nutrient recovery, valuable catholyte production and bioelectrochemical systems (MECs and BECs) devoted to nutrient recovery are described. Finally, as a key factor, the real implementation of BESs and the most important attempts to scale up the technology are described in detail. A summary of the possible improvements that can be made by implementing key factors such as electrodes, separators/membranes, design and scaling up is presented.

## 2 Human Urine Properties

**Figure 2:**
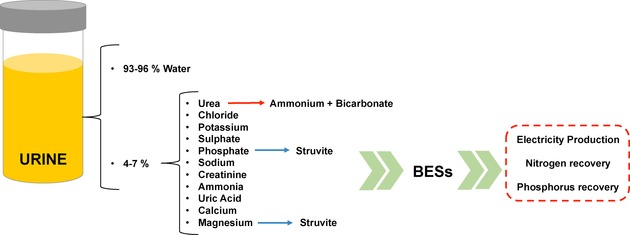
Composition of human urine and potential application as BESs feedstock. Schematic reported considering Refs. [15] and [27].

Human urine is a fluid generated by the kidneys and it has an amber transparent colour. The kidneys work as a filter and sieve the blood removing excess water and soluble waste.^26^ Urine is composed mainly of urea, chloride/potassium/sodium inorganic salts, ammonia, creatinine, organic acids and various toxins and products caused by the breakdown of haemoglobin. It is important to note the presence of urobilin, which is the cause of the characteristic urine colour. Moreover, urine consists of 93–96 % of water (Figure 2).^27^ Urine composition varies widely, and this is based on factors such as lifestyle, physical condition, environmental conditions (e. g. temperature, altitude on sea level, season, humidity, etc) as well as diet.

Considering a daily production of roughly 1.5–2 L of urine per person and a world population of 7.7 billion people, the daily global production of urine is estimated to be in the range of 1.16‐1.54×10^10^ L which equates to 4.22–5.62×10^12^ L a year. The measured dry matter in urine is 4.7–10.4 g L^−1^, which is equivalent to a urine solids loading rate between 57 and 64 g cap^−1^ day^−1^.^28^ Urea is a major component with over 50 % in weight of the organic solids.^11^ The chemical composition was measured by Strauss in 1985^29^ who found that the elemental composition of the dry urine solids is mainly composed by nitrogen, carbon, phosphorous and potassium. Particularly, the elements found were 14–18 % N, 13 % C, 3.7 % P, and 3.7 % K. In concentration, the main elements in urine counted for 6.87 g L^−1^ (C), 8.12 g L^−1^ (N), 8.25 g L^−1^ (O), and 1.51 g L^−1^ (H).^30^ When compared to faeces, urine exhibits the highest proportion of N (90 %), P (50–65 %), and K (50–80 %).^31^ The amount of nitrogen excreted is generally above 1 L g cap^−1^ day^−1^ (depending on nitrogen uptake) and the form in which it is excreted is mainly urea (between 75 % and 90 %).^32^ Another important nitrogen‐rich molecule excreted is creatinine with values measured in the region of 1.4‐1.9 g cap^−1^ day^−1^.^33^ Phosphorous concentration in urine mainly comes in the form of phosphate with concentrations varying between 0.2 g L^−1^ and 2.5 g L^−1^ whereas the concentration of potassium ranges between 0.4 g L^−1^ and 2.6 g L^−1^ and calcium between 0.03 g L^−1^ and 0.23 g L^−1^. The reported COD levels in urine range between 8–17 g L^−1^.^11^

As already mentioned, human urine is responsible for 10 % of COD, 75 % of N and 50 % of P in civil wastewater.^12^ With the data presented above, source‐separated wastewater treatment represents an important pathway to pursue, especially when considering recovery of nutrients. When human urine first leaves the body it has a slightly acidic pH in the range of 5.5 and 7.^34^ It is well known that after a short period of a few hours, depending on temperature, the pH shift towards alkaline values between 8.5 and 9.5. This transformation is naturally driven by the presence of the urease enzyme that breaks down urea to produce carbon dioxide and ammonia/ammonium ions.

Human urine contains a range of ions and therefore it has high solution conductivity. A recent study, considering the urine of a single individual but taken at different times, showed that the urine had a variable solution conductivity between 7–20 mS cm^−1^.^35^ This value tripled after one‐day of operation measuring 21–63 mS cm^−1^.^35^ After the natural hydrolysis of urea, the solution conductivity tends to become much higher as the urea breaks down into ammonium ions. As previously mentioned, BESs are mainly limited due to the high ohmic losses of the electrolyte and therefore the utilisation of an electrolyte containing organic molecules such as urine ‐that is highly conductive‐ is beneficial for the overall electrochemical performance.

## 3 Bioelectrochemical Systems Fed with Urine

The improvements in performance are discussed herewith, involving MFCs that utilise urine as feedstock. Particular attention is given to two types of MFC design, one membrane‐based and the other membraneless. Urine has also been used in other bioelectrochemical systems for the recovery of valuable nutrients such as ammonia and phosphate. Particular attention is given to the natural transformation of nitrogen and phosphorous compounds during urine hydrolysis. Moreover, the catholyte production by the extraction of valuable salts from urine is presented. Other BESs for extracting and recovering ammonia (microbial electrosynthesis cell) and concentrating nitrogen and phosphorous containing compounds (microbial concentration cell) are presented.

### 3.1 Microbial Fuel Cell Powered by Urine for Electricity Production

Two main urine‐fed microbial fuel cells designs have been presented so far in the existing literature. Single chamber membrane‐based urine‐fed MFCs^16^ employ a polymeric membrane as separator between the anode chamber and the cathode. This design of MFC has also been implemented substituting the expensive polymeric membrane with a cheaper ceramic separator. A second design based on a membrane‐less urine‐fed MFC was introduced in 2013 by Santoro *et al*.^36^ Later on, Walter *et al*. also reported a membrane‐less set‐up based on the self‐stratification of urine within a column.^37^ In this case, the stratification of urine allows the bottom of the column to be in complete anaerobiosis and the top of the column to be anoxic or aerobic. In this section, advances in these two directions are described.

**Figure 3:**
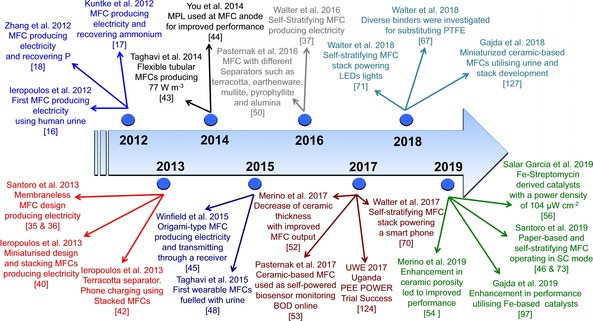
Historical overview showing the timeline with major achievements achieved in microbial fuel cells fuelled with urine

The historical overview of the development of MFCs fuelled with human urine is shown in Figure 3 where the improvements are highlighted.

#### 3.1.1 Single Chamber Membrane‐Based Microbial Fuel Cell Treating Urine

Use of urine as feedstock in MFC systems has gained significant attention in recent years, in part due to its abundance, composition of suitable nutrients for microbial growth as well as its high conductivity.^38^ The major components of microbial fuel cell include the cathode, anode and the membrane separator. Earlier reports showed that over 60 % of the cost of MFC systems was due to the ion exchange membrane.^39^ For long‐term commercial viability of MFC systems, cheaper materials would be required.

The first MFC utilising urine was based on acrylic compartments of 25 mL for anode and cathode separated by a polymeric cation exchange membrane.^16^ This experiment has been in operation for over 11 years and highlights the value of using urine as a substrate. In fact, the current increased substantially each time that the MFCs were fed with urine.^16^ During the same year, Zang *et al*. also produced electricity from urine using an MFC with a proton exchange membrane (PEM, GEFC‐10 N, China).^18^ In this case, the power generated ranged between 0.1 mW and 0.325 mW and the urine utilised was subject to pretreatment for reducing the phosphorous and nitrogen load. The same year,^17^ a double chamber MFC was employed not only for producing electricity but mainly to recover ammonia. In this study, a cation exchange membrane (Nafion 117) allowed for ammonium ions to migrate to the cathodic chamber, be transformed to ammonia and recovered by stripping.

In 2013, Ieropoulos *et al*. presented two novel miniaturised MFCs with anodic chamber volumes of 1.4 mL and 6.25 mL.^40^ In this case, a polymeric cation exchange membrane was used for separating anodic and cathodic compartments. According to this configuration, 48 MFCs were then stacked and a power of 1.5 mW was recorded.^40^

Ceramic membranes working as separators in MFCs provide the opportunity to use inexpensive and sustainable materials for waste treatment and energy generation, as well as *in‐country* production, since it is naturally available across the globe. Ceramics also carry the dual function of being the structural material for the cell chassis.^41^ The advantages of utilising ceramics as separators in MFCs are: i) lower cost compared to polymeric membranes, ii) natural availability; iii) chemical and thermal stability; iv) robustness for long terms operations; v) ease of adaptation with different designs and at different scales; iv) catholyte production.

In 2013, a stack of twelve MFCs having terracotta separators were tested using human urine with the peak power measured as 2 mW.^42^ This stack was able to charge a basic mobile phone.

**Figure 4:**
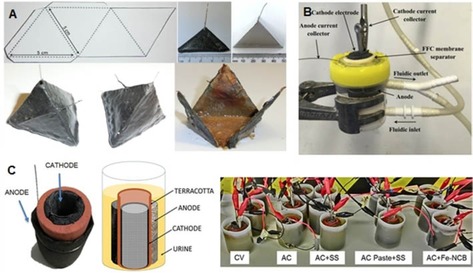
Different air‐breathing single chamber MFC set‐up fed with human urine for electricity production: A) tetrahedron paper based MFC^[45]^, b) fine fire clay‐based MFC^54^ and C) terracotta clay‐based MFC^[55]^. Figure A) adapted from Ref. [45]. Published by The Royal Society of Chemistry. CC BY 3.0. Figure B) adapted from Ref. [54], Elsevier, under licence CC BY 4.0. Figure C) adapted from Ref. [55], Wiley and Sons Inc., under licence CC BY 4.0.

For MFCs with conventional membranes, Taghavi *et al*.^43^ proposed totally flexible tubular MFCs fed with urine using Nafion membranes that achieved high volumetric power density (77 W m^−3^). You *et al*.^44^ utilised urine‐fed MFCs with cation exchange membranes and an empty anode volume of 6.25 mL. In this study, carbon veil or carbon cloth modified using micro porous layer (MPL) based on carbon black/PTFE was used for improving the anode and the overall power output by 35–50 % compared to the untreated anode material.^44^ Origami‐type MFCs fuelled with urine were also presented in 2015 (Figure 4A).^45^ This novel MFC configuration (15 mL empty volume) with a paper‐based separator achieved a peak maximum power of 50–60 μW. Six MFCs were then stacked, connected to a power management system (PMS) and provided enough energy to broadcast messages from a transceiver over 24 hours periods.^45^ More recently, this type of MFC, operating with human urine, was also investigated in supercapacitive mode with maximum power output of 1.38 mW (0.092 mW mL^−1^).^46^

Other unconventional membrane materials explored include fully biodegradable MFCs fed with urine.^47^ In this work the anodic chamber had a volume of 8 mL and was composed using a hard polylactic acid frame, a cation exchange membrane made of natural rubber and egg‐based open‐to‐air cathode that was coated with a lanolin gas diffusion layer to avoid leakage.^47^ A stack of 40 MFCs was tested for over 6 months and was able to power applications.

Taghavi *et al*. utilised the MFCs described before,^43^ using carbon fibre sleeves (anode), Nafion tubing (separator), carbon fibre sleeve (cathode) and stacked 24 MFCs around the legs of an individual. These wearable MFCs were fed with urine and were capable of producing enough power to transmit wirelessly.^48^ Fast prototyping using 3D printed moulds allowed the development of miniaturised MFCs utilising urine.^49^

Different types of ceramic materials have been tested within MFC systems including terracotta, earthenware, mullite, pyrophyllite and alumina.^50^, ^51^ These MFCs were fed with a mixture of urine and activated sludge. For instance, a comparison of the iron‐rich terracotta with an open porosity of 9.1 % and earthenware with an open porosity of 16.6 % showed that the less dense earthenware material generated higher power output,^41^ meaning that porosity plays the major role. A similar study which compared power outputs by various ceramic materials including earthenware, pyrophyllite, mullite and alumina showed that pyrophyllite and earthenware gave the best performance with power densities of 6.93 and 6.85 Wm^−3^, respectively, whilst mullite and alumina produced lower power densities of 4.98 and 2.6 Wm^−3^, respectively.^50^ The impact of the wall thickness of fine fire clay on power output and catholyte generation was evaluated by Merino Jimenez *et al*.^52^ The results showed that power generation decreased with increasing wall thickness, and as such the MFC with the thinnest membrane gave the highest power output of 2.1 mW compared to 1.3 mW produced by the MFCs with the thickest ceramic membrane. These results indicate that various properties of ceramics can all have significant impact on power output. Therefore, a combination of different factors that can aid performance can be incorporated into the manufacturing of the ceramic materials. One example of this is the incorporation of cation exchanger such as montmorillonite, which has been reported to enhance MFC performance both in terms of power output and coulombic efficiency.^41^ Ceramic‐based MFCs were also used by Pasternak *et al*. as a self‐powered biosensor for online monitoring of COD. Water and urine were used as COD source and the sensor was able to emit a signal when the COD concentration was higher than a pre‐defined threshold.^53^

Further advances in single chamber MFC fed with urine and possessing ceramic separators have been achieved in the last 2–3 years especially for the improvement of anode and cathode electrode materials incorporated in small‐scale MFC units. The power output by fine fire clay‐based MFCs was increased by 64 % only by changing the ceramic properties to higher water absorption, reaching up to 1 mW of power (Figure 4.B).^54^ In a similar small‐scale MFC made of terracotta, the output was reported to reach 2.19 mW with the use of an iron‐based catalyst on the cathode (Figure 4.C).^55^ These approaches allow the multiplication of small‐scale ceramic units into multi‐modular stacks increasing efficiency of scaled‐up systems. In 2019, Salar Garcia *et al*. operated ceramic separator MFCs fed with urine and used Fe‐streptomycin derived materials as a cathode catalyst and reached a maximum power density of 104 μW cm^−2^ which is the highest ever recorded for MFC treating a real human urine.^56^

Recently, it was shown that the number of pathogens was reduced significantly within MFCs operating with urine. Particularly, *Salmonella enterica serovar enteritidis*,^52^ *Salmonella enterica serovar Typhimurium*, *Pseudomonas aeruginosa* and *Staphylococcus aureus* 38 were subject to several log‐folds reduction and this was partially due to the increase in pH of the solution, but also due to the antagonism with the constituent electroactive communities.

#### 3.1.2 Membraneless Microbial Fuel Cell Treating Urine

As already mentioned, MFCs can be categorised in terms of the presence or absence of a membrane. In membrane‐less bioreactors, although the cathode and anode have different bioelectrochemical reactions, both electrodes share the same electrolyte. There is a range of setups reported in the literature that do not employ membranes but use glass beads, glass wool or sediments as separators.^58^, ^59^ In such systems, the electrolyte travels from the anode towards the cathodic part, or the other way around, therefore the electrodes share the same electrolyte (hydraulic continuum). The first membrane‐less design running under real condition was a 3.1.2. benthic type.^60^, ^61^, ^62^ Benthic MFCs have their anodes buried in the sediments and cathodes floating above them, in the water column. However, one could argue that such systems would not be a true membrane‐less design because of the presence of a physical separator limiting the diffusion of ions from one side to the other. The first membrane‐less MFC that did not have any physical material (i. e. sediment particles) separating the electrodes was reported by Liu *et al*. in 2004.^63^ In this work, the Authors reported that the removal of the polymeric membrane and the direct exposition of the cathode to the electrolyte solution were beneficial for improving the electrochemical output.

The first membraneless single chamber MFC treating urine was shown by Santoro *et al*. in 2013.^35^, ^36^ This was a simple set up, using a glass jar (volume 125 mL) modified with a lateral hole to accommodate an air‐cathode. Here power curves were measured over time and peak power decreased significantly from day 1 to day 42.^36^ Different feeding cycles were tested, and the COD degradation was 60–75 % (4‐days), 35–60 % (2‐days) and 25–40 % (1‐day). Ammonium ions were also measured and the concentration increased from less than 1000 mg L^−1^ to over 4000 mg L^−1^ after 1 day operation.^36^ Rapid precipitation of inorganic salts on the cathode due to the pH shift towards alkaline values was the main cause for the decrease in performance over a relatively short period of time.^35^ These two studies identify an important problem, which is the building of inorganic fouling on the cathode. Previously, it has been shown that cathode scaling leads to a rapid decrease in performance.^64^, ^65^ Therefore, for long‐term implementation, single chamber membraneless air‐cathodes might not be a suitable route to undertake.

The real implementation of MFC technology implies stacking a plurality of units, grouping the units into series and parallel electrical connections, and having a homogeneous flow distribution within the stack. Therefore, it is important for each MFC to have a simple design allowing easy replication and multiplication. Based on this, and to simplify a collective system, the self‐stratifying membrane‐less microbial fuel cell (S‐MFC) was developed.^37^ The structure of S‐MFCs resembles benthic MFCs in that there is a plurality of anodes positioned in the anoxic layers of an environment and a plurality of cathodes positioned in the oxic layer of the same environment. However, in S‐MFCs there is no material between the anodes and cathodes, which instead share the same electrolyte and are usually a distance of 5 mm apart. This type of design exploits a phenomenon observed in any liquid column colonised by life: the chemical and biological stratification of the column under biological activity. So although there is no physical material between the anode and the cathode added/found at the start, the growth of different biofilms on the anode and cathode, allows for a redox potential difference to be established that maintains a redox gradient.

In undisturbed natural environments (e. g. sediments, lakes), chemical gradients will develop under the activity of biological populations and become naturally divided in horizontal layers, each one characterised by specific bio‐chemical conditions (i. e. redox state of chemical elements, type of dominating metabolic activity). Along with microbial mats, lakes protected from wind mixing are good examples of self‐stratifying ecosystems as they display seasonal horizontal biogeochemical stratification of the water body due to biological activity.^66^

**Figure 5:**
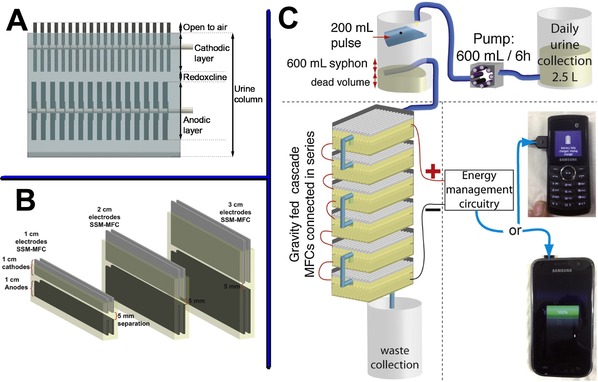
Illustration of a membrane‐less self‐stratifying microbial fuel cell: within the same urine column, an array of cathodes is placed above an array of anodes (A). Scaling down schematic of S‐MFC (B). MFCs set up cascade utilised for powering telecommunication devices (C). Figure B) was rearranged from Ref. [68], Elsevier, under licence CC BY 4.0. Figure C) was rearranged from Ref. [70], Elsevier, under licence CC BY 4.0.

The idea behind S‐MFC was to exploit the abovementioned self‐stratification phenomenon to develop a design of membrane‐less MFC to treat urine. The principle is to employ the capacity of microorganisms to structure a urine column into two layers ‐ an oxic and an anoxic. Therefore, this exploits the capacity of microorganisms to maintain two environments with different redox potentials, both separated by a redoxcline, thus forming a sort of “autogenic and transient membrane”. Having a self‐generated membrane of a few millimetres thickness (2 mm≤*x*≤8 mm) enables the use of multiple cathodes and anodes sharing the same electrolyte, with an array of vertical anodes placed in the lower reduced layer of the urine column, and an array of vertical cathode placed in the above oxic layer (Figure 5A). Therefore, maximising the electrodes surface area enables the densification of the electroactive reactions whilst keeping short diffusion distances, which result in high power density levels. Walter *et al*. demonstrated in 2016 that (1) the “transient chemical membrane” was sufficient to prevent losses from an electron flow between the anodic and cathodic layers, and (2) the reactors were scalable in length and width with minimal efficiency losses.^67^, ^68^, ^69^

Further studies have framed the operating conditions and design parameters of this type of MFC when fuelled with human urine. In terms of material, anodes were made of carbon veil and cathodes were made by hot pressing an activated‐carbon (AC) and polytetrafluoroethylene (PTFE) mixture (80 % AC; 20 % PTFE; 280 °C) onto a stainless‐steel 316 mesh acting as the current collector.^67^ In terms of scaling, it has been shown that a module can be downscaled to ≈4 cm urine column before showing losses in performance due to oxygen diffusion into the anodic layer (i. e. 0.75 mm deep cathodic layer) as shown in Figure 5.B^63^. In terms of operating conditions, the cathodes need to be at least partially exposed to atmospheric oxygen for the S‐MFC to function. More precisely, the optimal configuration is to have 3/4 of the cathodes immersed in the liquid with 1/4 exposed to air.^69^

This design has been developed further and is core to recent examples of practical applications in particular autonomous MFC‐systems fuelled by urine and powering low‐power applications such as telecommunication devices^70^ (Figure 5C), LED‐lighting systems^71^ or microcomputers.^72^ The first practical demonstration was made by assembling a full autonomous system tailored for a single user (≈2.4 L urine per day) and able to charge a modified Samsung GT−E2121B (ca.150 mAh lithium battery). A single cascade of 6 modules electrically connected in series produced a continuous electrical output of 2.5 V at 40 mA (100 mW), and was able to power 3 h of continuous phone communication every 6 h, with as little as 600 mL urine every 6 h.^70^

Beside urine treatment and power generation, this type of design was also applied to the development of internal self‐powered supercapacitive MFCs fed with human urine.^73^ Supercapacitor MFCs (empty volume 550 μL) were shown to produce a peak power of ≈1.20 mW (≈2.19 mW ml^−1^) for a pulse time of 0.01 s that decreased to ≈0.65 mW (≈1.18 mW ml^−1^) for longer pulse periods (5 s). Moreover, these microbial supercapacitors demonstrated relatively stable operation over 44 h with ≈2600 recharge/discharge cycles.^73^

### 3.2 Recovery of Nutrients Using Bioelectrochemical Systems Fed with Urine

#### 3.2.1 Natural Transformation of Nitrogen‐ and Phosphorous Containing Compounds During Urine Hydrolysis

As mentioned above, human urine has an initial pH that varies between 5.5 and 7.^34^ When urea hydrolysis occurs, the pH shifts towards more alkaline values between 8.5 and 9.5.^34^ Human urine from healthy individuals is fairly stable and unlikely to contain any microorganisms. However, when urine is stored in non‐sterile conditions, contact with bacteria is unavoidable. Ureases mainly from bacteria catalyse the hydrolysis of urea in stored urine to ammonia/ammonium and bicarbonate. Urease‐positive bacteria including eukaryotic and prokaryotic organisms are always present in toilet systems, since they are ubiquitous in soil, aquatic systems and also in human intestines and infected urinary tracts.^74^ The overall urea hydrolysis reaction can be written as follows [Eq. (1)]:

(1) $$\mathrm{NH}{}_{2}\left(\mathrm{CO}\right)\mathrm{NH}{}_{2}+2H{}_{2}O\to \mathrm{NH}{}_{3}+\mathrm{NH}{}_{4}{}^{+}+\mathrm{HCO}{}_{3}{}^{-}$$

Due to the ammonia release, this reaction causes the pH to increase up to 9.5 and triggers the precipitation of phosphorous, calcium and magnesium to form struvite (MgNH_4_PO_4_⋅6H_2_O), hydroxyapatite (HAP, Ca_5_(PO_4_)_3_(OH)) and occasionally calcite (CaCO_3_) [Eqs. (2)–(4)].^75^, ^76^

(2) $$\mathrm{Struvite}:\;\mathrm{Mg}{}^{2+}+\mathrm{NH}{}_{4}{}^{+}+\mathrm{PO}{}_{4}{}^{3-}+6H{}_{2}O\to \mathrm{MgNH}{}_{4}\mathrm{PO}{}_{4}.6H{}_{2}O$$

(3) $$\mathrm{Hydroxyapatite}:\;5\mathrm{Ca}{}^{2+}+3\mathrm{PO}{}_{4}{}^{3-}+\mathrm{OH}{}^{-}\to \mathrm{Ca}{}_{5}\left(\mathrm{PO}{}_{4}\right){}_{3}\left(\mathrm{OH}\right)$$

(4) $$\mathrm{Calcite}:\;\mathrm{Ca}{}^{2+}+\mathrm{CO}{}_{3}{}^{2-}\to \mathrm{CaCO}{}_{3}$$

In fresh urine, most of the nitrogen is present as urea. During hydrolysis, urea is broken to ammonia/ammonium, which accounts for most of the nitrogen in stored urine. With the elevating pH (around pH 9 after completion of urea hydrolysis), the concentration of ammonia increases. Ammonia is volatile and can easily escape to air, resulting in losses and lower nitrogen recovery efficiency (if nutrient recovery is a requirement). Another form of nitrogen in stored urine comes in the form of struvite (or magnesium‐ammonium‐phosphate, MAP) although the maximum amount of ammonium included in struvite is less than 1 % of the total ammonia in source‐separated urine.^76^

Almost all phosphorous (between 95–100 %) in fresh urine is in phosphate form, which is soluble.^12^ When pH increases as a result of urea hydrolysis, a portion of dissolved phosphorus then precipitates as struvite or hydroxyapatite. In undiluted urine, about 30–40 % of the soluble phosphate is converted to the precipitates,^77^, ^78^ although this can vary depending on the composition of urine. In modern central wastewater treatment systems using sewerage pipes connected to flushing toilets, struvite often causes pipe blockage, which incurs additional cost for cleaning or replacement. In many cases, other elements in use at wastewater treatment plants such as pumps, valves, centrifuges and aerators are also subject to fouling due to struvite deposits.^79^

The changes in the composition of undiluted urine during storage following urea hydrolysis and precipitation are shown in Table 1.

**Table 1:** Ions, compounds concentrations, physico‐chemical characteristics of fresh and stored urine according to references.^15^, ^79^, ^80^

	Average values of stored urine [mg L^−1^]			Average values of fresh urine [mg L^−1^]
Ref.	[15] [1]	[79]	[80]	[15]
Total nitrogen	9200	n.r. ^[2]^	8600	9200
Total ammonia‐N	8100	2390	n.r.	480
Ammonia NH_3_−N	2700	n.r.	431	0.3
Urea	0	n.r.	n.r.	7700
Total phosphate‐P	540	208	700	740
Calcium	0	16	106	190
Magnesium	0	<5	78	100
Potassium	2200	1410	1890	2200
Total carbonate	3200	n.r.	n.r	0
Sulphate	1500	778	1180	1500
Sodium	2600	1740	2410	2600
Chloride	3800	3210	3800	3800
Alkalinity	490	n.r.	n.r.	2.2
COD	10000	4500^3^	9000	10000
pH	9.1	8.69	n.r.	6.2

[1] Simulated values. [2] Not reported. [3] Dissolved COD

### 3.3 2 Catholyte Production During Microbial Fuel Cell Operations

Catholyte accumulation in previously empty air breathing cathode compartments is a combination of passive and active processes governing the quantitative and qualitative aspects of the formed liquid filtrate. The passive transport is dependent on the hydraulic pressure and the osmotic diffusion across the membrane, while the active processes are dependent on the current flow of the system. Active processes include the oxygen reduction reaction (ORR) occurring in the cathode electrode producing H_2_O or OH^−^ and the electroosmotic drag produced when the MFC is generating current, where the cations that migrate from the anode to the cathode drag water molecules along into the cathode compartment.

The ORR determines the physico‐chemical properties of the catholyte by forming OH^−^ as a product of the peroxide pathway^81^ leading to local alkalisation of the cathode where the increase in current flow will lead to higher pH.^82^, ^83^ It was shown recently that the shift between the acidic ORR pathway and alkaline ORR pathway occurs around pH 11.^84^, ^85^ When protons or cations move through the membrane, water molecules accompany them or are actually “dragged”. This phenomenon (well‐known as electroosmotic drag) was first described as charge induced flow by F.F. Reuss through a ceramic plug^86^ and is well‐studied primarily in the proton exchange fuel cells.^87^, ^88^ In MFCs, cation species move through the membrane and accumulate in the aqueous cathodic chamber resulting in increased solution conductivity and pH.^89^ In air breathing cathodes, the electroosmotic drag was first observed in MFCs by Kim *et al*., as the net water loss through the membrane that varied according to external resistance,^90^ that later led to the demonstration of the catholyte being accumulated as a result of the electrical current in wastewater operated MFCs.^82^, ^83^, ^84^, ^85^, ^86^, ^87^, ^88^, ^89^, ^90^, ^91^ The composition of the catholyte primarily depends on the type of anolyte (feedstock).^92^, ^93^, ^94^ Therefore, in urine operated MFCs, this opens new opportunities for nutrient recovery and recycling of elements suitable for fertilisers or irrigation. Changes of anolyte composition can affect struvite precipitation^95^ by increasing the pH and conductivity of the catholyte. Due to the high pH achieved, catholyte from MFC urine was able to drive electro‐coagulation that can be used for removing metal pollutants, such as copper, zinc and iron from aqueous solutions^96^ and prevent biofouling in long term operation.^97^ In a recent study, Merino‐Jimenez showed the effect of ceramic thickness on the ORR, catholyte production rate and quality, where the accumulation of cationic species in the produced catholyte from urine increased with the membrane thickness.^52^ The modification of ceramic properties such as composition and porosity can improve MFC performance while producing high quality catholyte^98^ directly from urine that can be used for practical applications such as disinfection.^99^ In urine fed MFCs, one of the main cations transported to the cathode is the ammonium ion that volatilises to ammonia due to the elevated local pH.^19^ Other cations moving to the cathode are calcium, magnesium, sodium, potassium and zinc.^19^ This is due to the ORR that switches to the alkaline pathway and the subsequent accumulation of the OH^−^ occurs.^100^ By enhancing current generation through ORR catalysis, driving the water extraction, pH and ion splitting, the MFC is able to filter urine and extract water against osmotic pressure.^55^ Recovering useful resources from urine would help to transform energy intensive processes to resource production, and will create new opportunities for future technology development addressing the energy‐water nexus.

### 3.4 3 Microbial Electrolysis Cell (MEC) Using Urine for Nutrient Recovery

As previously commented, most nitrogen (N) in domestic wastewater comes from the urine fraction (up to 75 %) in the form of urea.^12^ Following enzymatic hydrolysis, ammonia is formed with ammonium ions and ammonia making up to 90 % of the nitrogen in urine. Nitrogen‐based compounds must be removed from wastewater since their discharge to the public water network can facilitate eutrophication of water systems resulting in environmental issues. This process is energy‐intensive and time‐consuming posing significant challenges for the wastewater treatment companies. At the same time, the amount of nitrogen‐rich fertilisers synthesised has significantly increased in recent years in order to meet the agricultural needs.^12^, ^14^, ^101^

BESs such as MFCs have been successfully employed for nitrogen recovery and simultaneous bioenergy production.^17^, ^80^ However, the use of microbial electrolysis cells (MEC) instead of MFCs for ammonia recovery from urine can bring several other benefits. Economic analysis has shown that the removal and recovery of nitrogen from source‐separated urine might generate high revenue.^24^, ^25^, ^102^

**Figure 6:**
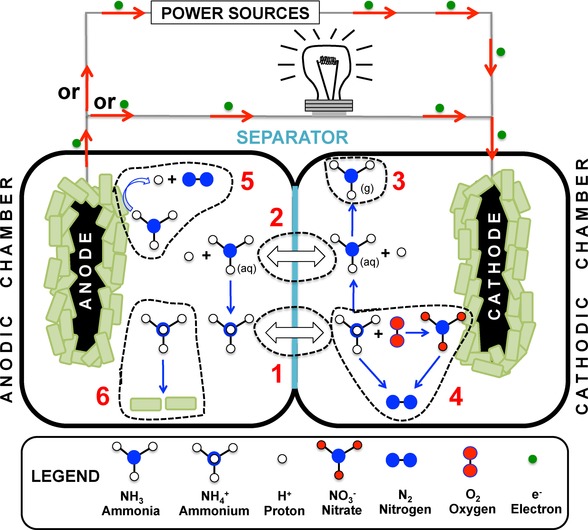
Ammonia removal mechanisms in MFCs and MECs. The figure was adapted with permission from Ref. [24], published by The Royal Society of Chemistry.

The transportation and transformation of ammonia and ammonium ions within BESs is summarised in Figure 6. Particularly: 1) ammonium ions transported through the membrane; 2) ammonia diffusion through the membrane; 3) the natural escape of ammonia gas from the system due to high pH; 4) ammonium ion biological oxidation using O_2_ and transformation in nitrogen gas on the cathode or in the electrolyte; 5) the hypothesis of anodic ammonia nitrification/denitrification forming nitrogen gas by microorganisms; 6) the utilisation of ammonium ions for building biomass on both anode or cathode.^24^

**Figure 7:**
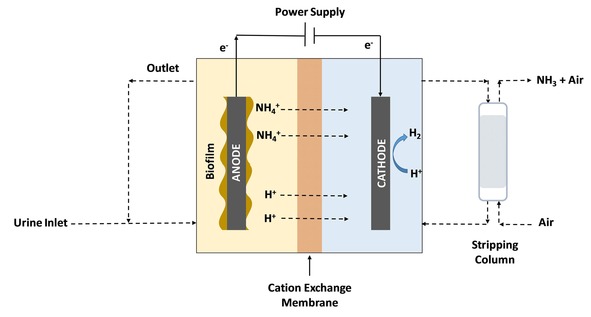
Urine‐fed MEC coupled to a stripping column for NH_3_ recovery.

Unlike MFCs, in a MEC the water produced in the cathodic chamber is reduced to hydroxyl ions and hydrogen. The presence of hydroxyl ions increases the pH of the cathodic compartment, which promotes the transformation of NH_4_^+^ into NH_3_. In addition to the current high value of the hydrogen produced, the presence of this gas reduces the necessity for aerating the cathode chamber, which is needed in MFC set‐ups designed for ammonium recovery. Moreover, the hydrogen gas produced can facilitate ammonia removal from the cathodic solution, which enables further removal of ammonium from the anolyte. Lastly, the current densities produced by MECs are higher compared with MFCs due to the applied external voltage, which also benefits the transport of ammonium through the membrane and also the removal rate.^103^, ^104^, ^105^ Figure 7 shows the schematic representation of a urine‐fed MEC coupled to a stripping module for ammonia recovery.

One of the first studies that focused on the use of MEC for treating urine and simultaneous hydrogen production and ammonium recovery was reported by Kuntke *et al*. in 2014.^19^ The Authors employed a double chamber MEC set‐up made of titanium, where the anode consisted of a piece of carbon felt and the cathode was decorated with a Pt‐based catalyst.^19^ Anodic and cathodic chambers were physically separated by a commercial cation exchange membrane (Nafion 117). Two individual reference electrodes were used to measure both anode and cathode potentials in a two‐electrode configuration. The current, applied voltage, electrode potential as well as the pH in both chambers were controlled at a constant temperature (30 °C). The system was inoculated with effluent from an MFC. After 6 weeks, the applied cell voltage increased from 0.6 V to 1.0 V and four different experiments were performed. One of the tests used synthetic wastewater as feedstock whereas the other two were fed with urine at two different feed flows. The last experiment was carried out also with urine, but the cathode solution was continuously replenished.^19^ Their results showed that the three experiments run with urine reached higher values of nitrogen removal than the system fed with wastewater. Moreover, it was observed that the continuous replenishment of the catholyte allows the system to reach more stable values of nitrogen removal. In this case, the ammonium removal rate was almost constant during the whole experiment whereas in the other three assays, the removal efficiency decreased over time. This system was able to reach a current density of 14.64 A m^−2^ coupled to a hydrogen production of 32 m^3^ H_2_ m^−3^ MEC d^−1^ and an energy input of 2.32 kWh m^−3^ H_2_,^19^ improving the results obtained previously by the Authors.^17^ The ammonium removal was reduced by 162.18 gN m^−^ d^−1^ from the anode and the COD decreased 130.56 g COD m^−2^ d^−1^. However, the amount of ammonium removal was still very low and the system for nitrogen removal via NH_3_‐stripping needs to be improved for large‐scale implementation of the technology. To overcome these challenges and enhance the ammonium removal rate, the Authors proposed two alternatives: i) to increase the migration of ammonium ions by optimising the COD removal and ii) to develop a more effective NH_3_‐stripping process to facilitate the ammonium reduction from the cathode and therefore facilitate the ammonium diffusion from the anode. The Authors have demonstrated feasibility in the use of the MEC technology for treating urine coupled to hydrogen production and ammonium recovery, however, further research is needed to improve its performance before large‐scale application can be considered.

Improvements to MECs utilised for recovery ammonia have been achieved by developing gas‐permeable hydrophobic membranes that enhance the collection of valuable ammonia.^106^, ^107^ Another important improvement in the system was achieved by recirculating hydrogen therefore diminishing the energetic requirements to efficiently recover ammonia.^108^ Recently, much attention has been given to a parameter named load ratio, which is the ratio between the current density applied, and the total ammonia nitrogen (TAN). This parameter is extremely important for optimising the current driven recovery of TAN from urine. Researchers are beginning to focus on optimising this parameter in order to enhance the overall recovery of ammonia and decrease energy consumption.^109^, ^110^

In 2015, Sotres *et al*.^111^ compared the ammonia recovery from a BES unit working first in MFC mode and then MEC mode. The BES consisted of a methacrylate double‐chamber reactor where anodic and cathodic compartments were separated by a commercial cation exchange membrane (Ultrex CMI‐7000). The anode was made of carbon felt mesh and the cathode consisted of a piece of stainless steel mesh. The system was fed with the liquid fraction of pig slurry and a stripping/absorption unit was integrated within the cathodic chamber for ammonia recovery from the catholyte.^111^ Their results showed that the BES exhibited higher COD removal rates and coulombic efficiency (CE) when the unit was working under MEC mode instead of MFC whilst the nitrogen flux through the membrane was similar. However, the Authors observed that by increasing the voltage applied when the system was working in MEC mode, the flux of nitrogen transfer to the cathode was higher, compared with MFC mode, reaching up to 25.5 gN d^−1^ m^−2^ when the voltage applied was 0.8 V. These results are in line with other research work previously published.^17^, ^103^ Ammonia recovery was around 2.4 times higher (94.3 %) than when the unit was working in MFC mode (38.8 %). These results might be related to the use of NaCl as a catholyte solution, which acidified this compartment promoting ammonia migration through the membrane. This acidic environment also facilitated ammonia recovery by the stripping/absorption unit when it is coupled to a BES system.

More recently, Cerrillo *et al*.^112^ studied the combination of anaerobic digestion (AD) and MEC in order to improve the quality of the effluent and recover ammonia by coupling a stripping/absorption unit. The anaerobic digester consisted of a cylindrical glass reactor where the temperature was controlled. The MEC was based on a double‐chamber unit fed with pig slurry where the compartments were separated by an Ultrex CMI‐7000 membrane. The anode was made of carbon felt and the cathode consisted of a piece of stainless steel mesh. The same configuration was previously used by the Authors to compare the improvement of combining AD with a BES working in MFC mode or as an MEC.^113^ The results of this work showed that the integration of an MEC with an AD unit allows the whole system to reach a stable COD removal of 46 % while recovering 40 % of ammonia. It was observed that the integration of an MEC not only helps to stabilise the AD against organic and nitrogen overloading but also to enhance the quality of the effluent and recover nitrogen in the form of ammonia.

A similar study was recently carried out where fermented urine containing easily degradable organics, produced higher current levels in MEC and higher COD degradation compared to fresh urine. Moreover, higher NH_4_^+^‐N removal was measured. This study suggests that a double stage anaerobic digester/MEC is a more effective option for increasing the organics degradation and improving the BES electrochemical output.^114^

Finally, two recent studies have carried out interesting microbiological analysis on the anodes of MECs operating with urine. In both cases *Phyla Firmicutes* and *Proteobacteria* were found on the anode materials.^115^, ^116^

#### 3.4.1 Nitrogen and Phosphorous Recovery Using Microbial Concentration Cell

Bio‐electroconcentration systems (BEC) are an alternative to MEC for nutrient recovery from urine. This hybrid technology comprises a microbial electrolysis cell and an electrodialysis cell. An electric field is generated from an external source and charged ions (cations or anions) selectively move from one chamber to another through specific polymeric exchange membranes. The main purpose of this type of technology is to concentrate anions and cations of interest within the central chamber.

**Figure 8:**
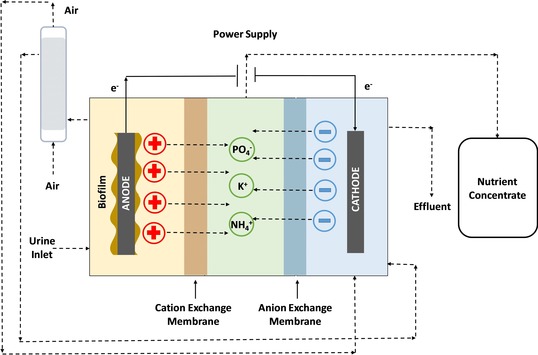
Depiction of the bioelectrochemical concentration system designed for nutrient recovery from synthetic urine. Adapted with permission from Ref. [20]. Copyright 2017, American Chemical Society

In this regard, Ledezma *et al*. (2017) reported the successful use of these devices for ammonia, phosphate and potassium recovery from synthetic urine.^20^ The three‐chamber reactor of 200 m^3^ of total volume was divided into three compartments named as anodic, concentrate and cathodic. Anodic and concentrate compartments were physically separated by a cation exchange membrane (CEM, CMI‐7000) whereas the concentrate and anodic compartments were separated by an anion exchange membrane (AEM, AMI‐7001). Anodic and cathodic electrodes consisted of plain graphite granules, however plain graphite rods were used as current collector in the anode, whereas a piece of titanium mesh was employed for the same purpose in the cathode. Anodic and cathodic electrodes were connected by an external circuit and a small amount of energy is applied to drive the hydrogen evolution (Figure 8). The energy balance of this system is positive so there is a net energy production. This energy is then applied on the system to facilitate the ion transfer through the membranes. The reactor was inoculated with a mix of acetate‐fed bioanode, urine and anaerobic sludge. The system was able to reach a maximum current density of 37.6 A m^−2^ at an applied voltage of 1.46 V, which is the highest value reported so far for nitrogen recovery in a MFC/MEC‐based system. The experiment was performed in triplicate and under these conditions, up to 7.18 kg of NH_4_^+^ m^−3^ day^−1^, 0.52 kg of PO_4_^−^ m^−3^ and 1.62 kg of K^+^ m^−3^ were removed and then recovered into a concentrate stream. Nitrogen‐rich solid compounds as pure NH_4_HCO_3_ crystals with a content of N measured in 17 % was recovered from the synthetic urine.^20^

The BEC system presented by the Authors opens the possibility of transforming a waste such as urine into a nitrogen‐rich solid compound, which can be reused as fertiliser. However, despite these promising results, further work is needed in order to improve the recovery efficiencies and reduce the energy consumption of the process, which will facilitate the practical implementation of this technology for treating urine.

Later on, in 2018 Jermakka *et al*. analysed the effect of the feedstock composition and the applied current density on the performance of a three‐chamber abiotic electroconcentration cell.^117^ The set‐up was similar to that used by Ledezma *et al*. (2017) but the cathode electrode composed of stainless steel mesh instead of plain graphite granules.^20^ The Authors prepared three different artificial urine‐based feedstock: i) urine without Mg nor Ca (previously precipitated with phosphate) (ACE), ii) urine without ammonium acetate (NO ACE) and iii) urine without acetate and higher amount of ammonium bicarbonate (ABC) due to the assumption that all acetate was digested into carbon dioxide.

Their results did not show significant differences in terms of current efficiency for ammonium transport through the CEM when different current densities or feeding compositions were applied (between 63–72 %). The energy consumption by the system varied according to the type of feedstock used, so when the electroconcentration cell was fed with ACE, the energy consumption was 30, 35 and 46 MJ kg N^−1^ at 40, 60 and 80 A m^−2^. However, 35, 39 and 47 MJ kg N^−1^ were consumed at 60, 80 and 100 A m^−2^ when the system was fed with ABC. This energy demand is quite high compared with electroconcentration cells using bioanodes (8.6 MJ kg N^−1^).^118^

More recently and based on the same concept, Freguia *et al*. (2019)^119^ reported the use of a bio‐electroconcentration cell similar to their previous work reported by Ledezma *et al*. (2017)^20^ but using an air‐breathing cathode. Unlike previously, in this work, real human urine was used to feed the system. The air‐breathing cathode consisted of a piece of carbon cloth coated with carbon‐based catalyst to facilitate the oxygen reduction reaction. The anode compartment was inoculated with effluent from a similar reactor.^20^ In this case, the energy produced by the oxidation of the urine organics in the MFC was sufficient to power the electrodialysis system and also, showed a net electrical current density produced of 3 A m^−2^. Coupled to the energy production, the system was able to recover 1.2 % N, 0.4 % K, 0.02 % P and 0.1 % S without any presence of heavy metals nor any external power supply. These results support the feasibility of combining MFC and MEC technologies to build a system able to treat human urine and recover nutrients.

#### 3.4.2 Nitrogen and Phosphorous Compounds Recovery from Human Urine Using Microbial Fuel Cells

The focus of researchers in the field has been about the recovery of ammonia through the utilisation of electrochemical and bioelectrochemical systems. This has mainly been driven by the need to find a system that could be competitive compared to the energy extensive Haber‐Bosch process for producing ammonia as fertiliser. Moreover, this process could remove nitrogen from wastewater avoiding complication in the treatment system as well as reducing costs. Less attention has been given to the recovery of phosphorous, which still accounts for 50 % of the overall P in domestic wastewater.^12^ As already mentioned, the natural increase in pH is due to urine hydrolysis and this favours the precipitation of phosphorous containing salts such as hydroxyapatite (calcium and phosphate) and struvite (magnesium, phosphate and ammonium). Generally, calcium and magnesium are the limiting ions for the precipitation of the salt.

Zang *et al*. enhanced the precipitation of struvite from hydrolysed diluted urine by adding magnesium sulfate and disodium phosphate since their concentrations compared to ammonium ions were low. A percentage of 95.6 % of struvite was found in the precipitate. The recovery of phosphate and ammonium increased after the treatment in MFCs.^18^

Santoro *et al*. monitored the ion concentration before and after treatment in microbial fuel cells fed with urine.^36^ It was found that ammonium ion concentration increased four‐fold due to urea hydrolysis. Sulfate and phosphorous concentrations tended to decrease while calcium and magnesium levels significantly dropped. The precipitate on the bottom was found to contain struvite, potassium struvite and hydroxyapatite.^36^ In another experiment, the decrease in P was monitored for MFCs fed with: 1) fresh urine, 2) wastewater from a treatment plant (WW), 3) wastewater with the addition of sodium acetate (WW+NaOAc) and 4) phosphate buffer and sodium acetate (PBS+NaOAc).^120^ The percentage of P removed was 40.1 % in the case of urine feeding that decreased to 15.7 %, 14.1 % and 2.5 % in the case of WW+NaOAc, WW and PBS+NaOAc respectively.

You *et al*. proposed a three‐stage struvite extraction process system.^72^ The first stage was composed of 4 MFCs in cascade fed fresh urine. The second stage was a precipitation process for recovering the struvite followed by the final stage in which urine from stage two was used as feedstock. In the precipitation process, MgCl_2_ was used for enhancing the precipitation and a removal of 20 % of NH_4_^+^‐N and 82 % PO_4_^3−^ was measured.^78^ The MFC cascade of the first stage and the third stage produced similar power. Interestingly, it was shown that the urea hydrolysis process was enhanced when urine was introduced into the MFC rather than natural hydrolysis and this might be due to the higher presence of bacteria.^78^

Merino‐Jimenez *et al*. increased the power production of MFCs fed with urine as well as the recovery of struvite by adding sea salts.^95^ As mentioned before, the struvite precipitation is limited by the divalent cations such as Ca and Mg that in urine have quite low concentration. Six different solutions were tested in MFCs: 1) neat urine (U); 2) urine and magnesium chloride (U+MgCl_2_); 3) neat urine stirred (US); 4) urine and artificial seawater (U+SW); 5) urine and SeaMix (U+SM) and 6) urine and DI water (U+DI). U+SM showed the highest performance and a recovery of struvite of up to 94 %. Moreover, the addition of SeaMix enhanced the pH, conductivity and chloride ions within the catholyte.^95^

In some of the previous reports, hydrolysed urine is used which potentially contains less P than fresh urine. Often, struvite is completely removed by adding magnesium chloride before being utilised in BESs. Struvite can affect electrode performance by precipitation on the electrode surface. This is negative for the anode because it decreases the biotic/abiotic interface and detrimental for the cathode because it increases the mass transfer resistance by inorganic fouling or deactivation of the cathode catalyst.

### 3.5 Pilot‐Scale Involving Bioelectrochemical Systems Using Urine

Pilot‐scale studies or trials refer to the pre‐testing of a new research instrument or technology. These can provide valuable insights that can inform the design of full‐scale systems and commercial products increasing the likelihood of success once deployed in real life scenarios. Additionally, pilot‐scale studies can help identify further research objectives and advance the research field. Over the past 8 years since the first utilisation of urine in MFC and MEC, several advancements have been achieved and the process for transformation of organics into valuable electricity or the transformation and recovery of nutrients has been optimised. This section will focus on scaling up the system and the various pilot level activities reported for MFC and MEC treating urine.

#### 3.5.1 Pilot‐Scale Microbial Fuel Cell Treating Urine

As previously mentioned, MFCs have been widely used for the treatment of urine and its conversion to usable electrical energy. Due to this ability, urine‐fed MFC systems have been deployed in recent years in numerous field trials around the world, exposing them to different environments and climates to observe their behaviour. Usually these systems are connected to urinals to channel “fuel” directly from the source so that it can be treated before diverting to the sewage or a soakaway. These pilot‐scale studies have played an important role in the advancement of the technology thus far and have positively contributed to development towards a full‐scale commercial product. The section below aims to review the pilot‐scale urine‐fed MFC systems reported to date.

**Figure 9:**
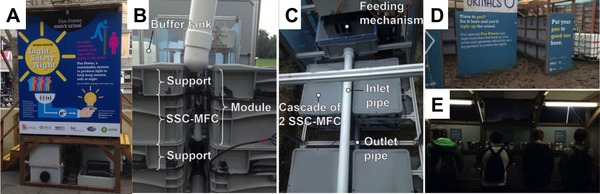
Pee Power field trial at the University of the West of England Frenchay campus 02–05/2015 (A). Installation at Glastonbury Music Festival 2016 (B). View from above the Glastonbury feeding mechanism (C). Image of the urinal from outside (2017) (D). Image of the urinal with music festival participants (E). Figure A) was adapted from Ref. [51], Elsevier, under licence CC BY 4.0. Figures B−E) were adapted from Ref. [71], Elsevier, under licence CC BY 4.0.

The first recorded pilot use of MFCs fuelled by urine was in March 2015 at the University of the West of England (UWE, Bristol) where a prototype of the Pee Power was trialled at the university's Frenchay campus (Figure 9A).^121^, ^122^ The prototype urinal was a collaboration between researchers at UWE and Oxfam, who aimed to use the Pee Power technology to light toilets in refugee camps that are often dark and dangerous, particularly for women. The urinal at Frenchay campus resembled toilets used in refugee camps by Oxfam to make the trial as realistic as possible. A stack of 288 MFCs distributed between eight plastic tanks was placed underneath the urinal and could be viewed through a clear screen. Each tank had a 24.5 L of urine capacity and contained 36 ceramic‐based MFCs with air‐cathodes as previously described.^121^ The MFC stack powered the internal lighting system of the urinal cubicle (4 LED domestic lights), which were triggered by an infrared motion sensor (also powered by the MFCs). The energy produced by the MFCs was stored in supercapacitors and discharged when motion was detected (i. e. a person entering the cubicle). This was initially a 3‐month trial (until May 2015), allowing for partial evaluation of the technology in the field, until it was carefully transferred to the Glastonbury Music Festival for the first time. Glastonbury is the UK's biggest music festival, bringing together ca. 250,000 people, and where the Pee Power urinal could be tested under different fluidic configuration and usage conditions (i. e. more frequent use). The original system described above was supplemented with four more MFC boxes (twelve in total) and was connected to a larger urinal structure that comprised 3‐troughs and could accommodate 10‐people at once. The total volume of the system was 330 L of urine and it powered 6 LED modules throughout each night.^123^ The chemical oxygen demand (COD) removal was >95 % for the campus urinal and on average 30 % for the Glastonbury urinal. The experience of the two field trials in 2015 informed the development of the next generation of improved performance MFC systems suitable for field use.

A year later, in 2016, a modified Pee Power system was installed at that year's Glastonbury Music Festival for three weeks (Figure 9.B‐9E).^66^ The aim of this trial was to field‐test different MFC designs and scale up, whilst keeping the footprint of the MFCs smaller than the 2015 version. Additionally, a passive feeding mechanism was designed that controlled the hydraulic retention time (HRT) during times of no usage (i. e. early mornings). This system employed self‐stratifying membrane‐less MFCs (S‐MFC) which allows the microorganisms to vertically self‐stratify across physicochemical conditions of any given water column (e. g. lake, or urine).^71^ This urinal had doubled the theoretical capacity in terms of users compared to the urinal used in 2015. The system consisted of a 12 S‐MFC module stack, a power management system (to harvest and distribute the energy) and a passive feeding mechanism (Figure 9.C).^71^ The system was able to power 6 commercially available Auralum (T8) LED tube lights which provided lighting in the urinal, enough for users to be able to read clearly the information posters placed inside the cubicle. Each LED tube light was modified to run at the desired 2.650 V DC voltage and was turned on for approximately 9 h 30 min per day. Compared to the 2015 Glastonbury field trial, the 2016 system achieved a 37 % higher COD removal.^71^

Since 2016, improved Pee Power systems have been installed overseas, in remote areas of developing countries, such as Kisoro, Uganda and Nairobi, Kenya. Both systems provided lighting for toilet blocks at boarding schools.^124^, ^125^ These field trials form a good indication that MFC technology can be incorporated into real life scenarios showing their potential as an off‐grid electricity production system.

#### 3.5.2 Microbial Electrolysis Cell Pilot‐Scale Treating Urine

**Figure 10:**
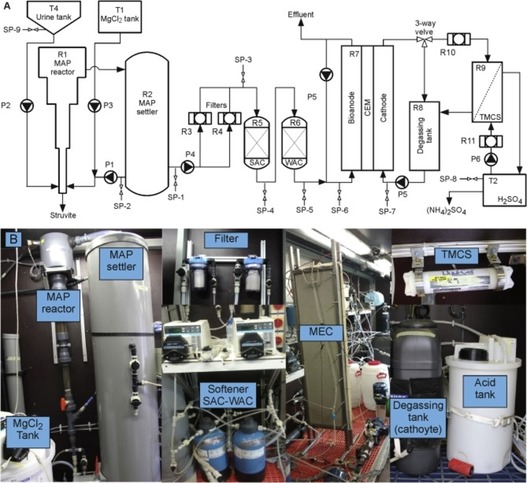
Flow scheme diagram (A) and pictures (B) of the scaled‐up MEC for nutrient recovery and energy production from urine.^126^ Arranged from Ref. [126], Elsevier, under licence CC BY 4.0.

In previous sections, laboratory scale MECs for nutrient recovery from urine have been described. However, due to the importance of scaling‐up BES for actual implementation, this section focuses on the successful scaling‐up of MECs. In 2017 Zamora *et al*.^126^ reported the first scaled‐up MEC for recovering both ammonia and ammonium nitrogen (TAN) from urine coupled to a pre‐treatment stage for phosphorus recovering. Thus, the process comprised two stages: i) the first one was the phosphorous recovery in the form of struvite by using a fluidised bed reactor (MAP) and ii) the second step focused on the urine treatment for TAN recovery by using a MEC unit (Figure 10).

Anodic and cathodic chamber were physically separated by a cation exchange membrane (Ralex) and catholyte and anolyte were continuously fed during 6 months. In this stage, the TAN was recovered by absorption in an acidic solution using a gas permeable hydrophobic hollow fibre membrane (TMCS). During the first stage, 94 % of phosphorous was precipitated as pure struvite and TAN was reduced by about 16 %. Regarding the second step, the system reached a current density of 1.9 A m^−2^ by applying a voltage of 0.5 V. Coulombic efficiency was around 70 % and the COD was reduced by 20 %. 31 % of the nitrogen present in the anolyte crossed the membrane and was totally recovered through the TMCS module. The energy needs for TAN recovery was around 4.9 MJ kg_N_^−1^, much lower than other electrochemical technologies. This study demonstrated the first scaled‐up MEC for successful nutrient recovery from urine. However, scaling up BES for TAN recovery poses several challenges due to the design of the treatment process as well as the limited COD removal. Therefore, it is crucial to select a suitable reactor design and materials, which will favour the conditions of the process. Finally, in order to improve the current density and therefore the TAN recovery, it would be beneficial to hydrolyse complex compounds present in urine such as proteins or amino acids previously to facilitate their degradation and therefore, the treatment capacity of the BES.

## 4 Summary and Outlook

Human urine is a waste with unique characteristics. Urine only makes up 1 % of domestic wastewater, yet it contributes 10 % COD, 50 % phosphorous and 75 % of the nitrogen found in the entire municipal wastewater.^12^, ^14^, ^15^ As discharge limitations in water bodies are becoming increasingly more stringent, nutrient removal and possibly recovery becomes an important activity. Diverting urine from the sewage as source‐separated, treating urine separately by transforming organics into valuable electricity and remove/recover nutrients seems the pathway to pursue. This topic has captured the attention of hundreds of scientists worldwide. The only technology available that is capable of producing electricity directly from a (circum)neutral organic concentrated waste is microbial fuel cells. Several reports on this have been presented in the last few years with continuous improvements in design, COD removal and power output. The power output was enhanced firstly by improving materials for anode and cathode electrodes. Particularly, the anode material conductivity has been enhanced as well as the biotic/abiotic interface. Cathode reactions have been improved by adding suitable cheap catalysts capable of accelerating the reduction of oxygen and being durable in harsh and contaminated environment. Separator properties are now better understood and adapted to the technological needs for enhancing proton transfer and preserving the catalytic activity. It has been shown that miniaturisation and multiplication is a valid pathway for enhancing overall power and scale up of the system.^127^ Similarly, in the case of S‐MFC, the reduction of “dead” space among electrodes can enhance the electroactive surface reducing the volume in which fermentation, a parasitic reaction, might occur. Moving from laboratory scale to pilot‐scale has its own challenges that are being overcome through trialling in real environments. However, from the electrochemical point of view, urine has high solution conductivity that is beneficial for reducing the electrolyte ohmic losses. This is of extreme importance for MFCs, which are traditionally ohmic limited electrochemical devices. Moreover, compared to domestic wastewater, urine has a pH which is slightly more alkaline, and this is beneficial for the oxygen reduction kinetics which need H^+^ or OH^−^ as reagents.

Bioelectrochemical systems represent a technology that utilises otherwise too‐wet‐to‐burn or diluted waste to generate relatively low current/power levels, with the additional benefits of producing valuable by‐products and recovering nutrients. Despite the multiple benefits of this platform technology, such features have been demonstrated at relatively low levels or rates to be competitive with existing power source technologies or fertiliser production processes, which have had far longer periods of development and funding. BES have to be low cost, durable and efficient and in order to further optimise their performance, significant effort has to be devoted to the appropriate selection of electrode and separator materials, optimal design and operational parameters, all of which play a critical role in the overall system performance.

With regard to anode materials, research should concentrate on developing materials possessing i) high surface area to increase the bacteria/electrode interface and reagents/products diffusion; ii) hydrophilicity for enhancing bacterial attachment, iii) high electrical conductivity, iv) corrosion resistance, v) environmentally friendly and low cost. In order to increase the performance of the current state‐of‐the‐art systems, existing commercially available carbonaceous‐based or metallic‐based anodes could be modified through doping the electrode surface with oxygen or nitrogen functional groups, carbonaceous conductive materials (e. g. carbon black, graphene, etc), electrochemically active polymers and transition metal oxides and nanoparticles.^128^, ^129^ Regarding the cathodes, as the oxygen reduction reaction (ORR) is sluggish in circumneutral pH, in order to improve the performance output, the most used air‐breathing cathode based on AC and PTFE mixture should be modified appropriately. Particularly, the cathode should be decorated with conductive carbonaceous materials (e. g. carbon nanotubes, graphene, carbon black, carbon nanofibers, etc.) for enhancing the electrode conductivity and the overall ORR. Moreover, the ORR could be enhanced by the integration of transition metal coordinated with nitrogen and carbon (M−N−C with M as Fe, Co, Ni, Mn, etc.) that have already shown superior catalytic activity and achieved the highest performance in MFCs treating urine;^56^ these catalysts have also shown durability in long term operations.^97^ In MEC, hydrogen evolution reaction (HER) is the main reaction occurring on the cathode. Noble metal use such as platinum or platinum‐derived materials should be avoided as this increases the cost significantly and also suffers from anion poisoning.

When it comes to membranes and separators, improvements should be driven in the direction of controlling porosity and therefore enhancing selectivity, decrease ohmic resistance and enhance robustness for long terms operations. These objectives should again be met by keeping low the cost of the separator or membrane or even lower it when possible.

Improving the design is another important challenge that has to be undertaken. While it is established that when area to volume ratio is high, higher power generation is achieved by reducing the “empty space”, which in turn optimises the operating anode area and the oxidation reactions occurring on it. The biggest challenge for scaling up the system would be to maintain scalable current/power produced despite multiplication of single miniaturised unit or when increasing the size of the electrodes. In this direction there is still space for further important improvements.

Being rich in nutrients, especially nitrogen under the form of ammonium/ammonia and phosphorous under the form of phosphate, urine is of extreme interest for the recovery of both nutrients for utilisation in agriculture as fertilisers. Ammonia production using traditional Haber‐Bosch process is energy‐intensive and therefore expensive. In parallel, the removal of nitrogen from wastewater under the form of nitrate and ammonium require more energy and often is complicated to modify and enlarge existing treatment plant. Moreover, in water treatment, ammonium and nitrate are transformed in nitrogen gas and therefore they are removed but not recovered as valuable products. Microbial electrolysis cell and bioelectrochemical concentration cells give important opportunities for recovering nutrients into value added products such as ammonia or ammonium bicarbonate. As opposed to MFCs, MECs and BEC utilise an external power source for promoting ion migration and nutrient recovery. Pilot studies have demonstrated an increase in the volume treated and indicate that commercial interests are present towards these technologies. Preliminary economic studies have shown the potential of these technologies to replace existing ammonia producing technologies.^24^, ^25^ Concerning phosphorous recovery, struvite has important properties and it can be utilised as slow‐releasing fertilisers. As opposed to ammonium, that converts to ammonia and therefore in gas phase, the transformation of phosphorous occurs through precipitation i. e. in the solid phase. This precipitation might be detrimental for the bioelectrochemical system covering the anode or the cathode electrode. In the majority of the studies presented in the literature, phosphorous is removed before being tested in BESs.

The existing literature on bioelectrochemical systems treating urine teaches us valuable lessons and provides us with several opportunities and pathways to pursue. In fact, urine can be used effectively as substrate for MFCs, MEC and BEC. Each of these BESs has its own characteristic: i) reducing COD, producing electricity and catholyte (MFCs); ii) reducing COD by consuming electricity and recovering ammonia gas under the form of ammonium sulfate (MECs); iii) reducing COD by consuming electricity and recovering solid ammonia bicarbonate (BECs). A multiple step system might be envisioned by integrating all three BES to take advantage of all of their specific characteristics. Improving BES design, electricity and nutrient recovery, durability and integrity of materials are the key aspects to follow towards commercialisation. Several other pilot‐scales with improved output levels are expected to be seen in the coming years.

## Conflict of interest

The authors declare no conflict of interest.

## Biographical Information

*Dr. Carlo Santoro got his BSc (2006) and MSc (2008) in Environmental Engineering at the Politecnico di Milano. He then moved to the University of Connecticut in 2009 obtaining his PhD in 2013. He studied microbial fuel cell for simultaneous wastewater treatment and electrical energy production. He moved to the University of New Mexico (UNM) in 2013 for his first Post Doctorate. He developed hybrid MFCs with microbial anode and enzymatic cathode. As second Post‐Doctoral experience, he worked at Nanyang Technological University developing VOCs detecting biosensors. In 2015, he moved back to UNM working on: i) low‐cost platinum‐free catalyst for oxygen reduction reaction; ii) supercapacitive bio‐electrochemical systems. From 2017 to 2020, he was Associate Professor in BioEnergy at UWE Bristol. He has been successfully awarded by USAMRDC, JCVI, B&MGF and NBIC (>0.7 M$). Till now, he published 85 manuscripts on highly ranked journals in 9 years with H_index_=32*.

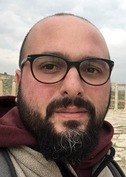

## Biographical Information

*Dr. Maria Jose Salar Garcia is a postdoctoral researcher at Bristol Bioenergy Centre (University of the West of England). She received her Ph.D in the Department of Chemical and Environmental Engineering (Technical University of Cartagena, Spain) in 2016 and was awarded with the extraordinary Ph.D award. After her Ph.D, she was funded by Seneca Foundation (Science and Technology Agency for the Region of Murcia) to conduct her research at Bristol BioEnergy Centre. Her research field focuses on the design and optimisation of Microbial Fuel Cells as well as the use of green solvents such as ionic liquids in membrane technology. These research lines have resulted in more than 40 scientific publications (h‐index: 13), 8 book chapters and 37 communications in both international and national congresses*.

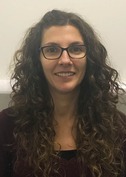

## Biographical Information

*Dr. Xavier Alexis Walter is currently a Senior Research Fellow at the Bristol Bioenergy Centre. He obtained his MSc in Biogeosciences and a PhD in Geomicrobiology at the University of Neuchâtel (Switzerland). He developed an expertise in stratified and complex microbial ecosystems and transferred this experience toward developing bioelectrochemical processes at the Bristol Bioenergy Centre. He specialised in self‐sustainable microbial electrochemical systems. These systems mimicking natural phenomena are implemented as processes generating power whilst simultaneously treating waste. In particular, his work focuses on self‐stratifying membrane‐less Microbial Fuel Cells that exploit the natural physicochemical stratification phenomena observed in liquid settings. This work has been implemented as pilot‐scale trials in informal settlements and refugee camps*.

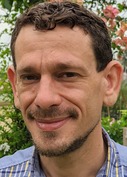

## Biographical Information

*Dr. Jiseon You is a Research Fellow in the Bristol BioEnergy Centre of the University of the West England (UK), which is led by Professor Ioannis Ieropoulos. She holds an MEng in water/wastewater treatment from the University of Science and Technology (Korea) and a PhD in microbial electrochemical systems from the University of the West of England (UK). Her research concentrates on MFC system performance improvement, integration of MFC technology into future living spaces and urban design. She is also interested in electrochemical resource recovery, system sustainability, and WASH (water, sanitation and hygiene)*.

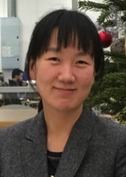

## Biographical Information

*Dr. Pavlina Theodosiou is a Postdoctoral Research Associate at Bristol Bioenergy Centre (UWE, Bristol) working on the research and development of the PeePower technology. Her project is funded by OXFAM and focuses on implementing the PeePower in refugee camps and slums in the developing world. She has a background in Biological Sciences (BSc) and a PhD in Bioenergy and Self‐Sustainable Systems. Her PhD was funded by the European Commission under the project EVOBLISS (FP‐7). For her thesis she was working with the robotic platform EvoBot, a 3D‐printer turned to robot, which she used as an automated Robot‐Chemostat for culturing and maintaining Microbial Fuel Cells (MFCs). The improved MFCs powered the 2018 edition EcoBot‐II and for this work she was awarded the “Best Biology Paper” at the 6^th^ Living Machines Conference*.

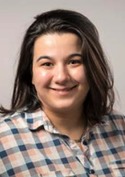

## Biographical Information

*Dr. Iwona Gajda is a Research Fellow and part of the team working on Urine‐tricity Project founded by the Bill and Melinda Gates Foundation, looking into clean energy recovery from waste through the MFC functionality as a direct power source for powering LEDs and as a filtration device for clean, purified catholyte that can be used for elemental recovery, carbon capture and disinfection. Her work with microalgae used in the cathode has indicated the feasibility of developing photo‐microbial fuel cells into the future. Her research interests include the design and architecture of MFC reactors and stacks, power improvement through improved materials, stacking and multiplication of MFCs for usable power levels and possible uses in robotics and sensing*.

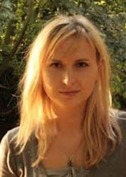

## Biographical Information

*Dr. Oluwatosin Obata is currently a Post‐Doctoral Research Associate (Dept. Chem. Eng.) at Newcastle University. Dr Obata's research is based on the three areas of bioenergy, environment and sustainability, using biotechnological approaches. His current research is on the Bio‐electrochemical systems (BESs) for material recovery from industrial wastewater, with special focus on zinc and copper recovery from steel making industries using various bio‐electrochemical approaches. He also has expertise in microbial ecology of various engineered systems such as Anaerobic digestion, BES systems, with the aim of optimizing the various electrochemical processes. He worked as a Research Associate at the Bristol Bioenergy Centre. His research was part of the Urine‐tricity project involving electricity generation from human urine and wastewater using BESs. He contributed significantly to the field trials of the technology in the UK and parts of Africa*.

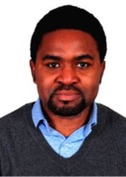

## Biographical Information

*Dr Jonathan Winfield holds a BSc Zoology degree (University of Bristol, 2007) and a PhD in Environmental Technology (University of the West of England (UWE), 2011) where he investigated microbial fuel cells (MFCs) and their potential for wastewater treatment. His is lecturer at UWE and has a particular interest in developing novel MFC component materials. He has investigated unusual applications for MFC technology including onboard energy supply for biodegradable robots, powering artificial muscles and origami alarm systems*.

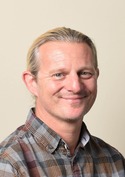

## Biographical Information

*Professor John Greenman is an Emeritus Professor at the University of the West of England, Bristol with a specialty in microbiology, biofilms, continuous culture and Microbial Fuel Cells. He has worked on continuous culture systems, including chemostats, turbidostats, thick and thin biofilm growth systems using perfusion systems. Greenman (with Ieropoulos) was one of the originators of the term Symbot to describe the symbiotic relationship between robots and biological entities such as biofilm‐electrodes, as exemplified through the EcoBot series of Robots. Greenman (with Ieropoulos) also discovered the advantages of small scale MFC over large chamber MFC and played an important role in developing a self‐powered, floating biosensor for online water quality monitoring and MFC stacks for treating urine. His work with MFC goes back over 20 years, and Greenman is now working on new applications of MFC, especially MFC integrated with microalgae using PMFC*.

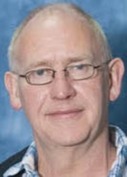

## Biographical Information

*Ioannis A. Ieropoulos is Professor of Bioenergy & Self‐Sustainable Systems and Founder and Director of the Bristol BioEnergy Centre, Bristol Robotics Laboratory, UWE. He has an interest in waste utilisation and energy autonomy and produced the EcoBot family of robots and RowBot, which have their own MFC microbiome and operate completely devoid of conventional power sources. He has been an EPSRC Career Acceleration Fellow (2010‐2015) and is currently a Bill & Melinda Gates Foundation grantee on the “Urine‐tricity/PEE POWER” project, advancing the MFC technology for sanitation improvement in Developing World Countries. He leads projects, focusing on robotics, biodegradable & functional materials, funded by the Leverhulme Trust, and the European Commission (FP‐6, FP‐7 and H2020) with a focus on living architecture. He has published >100 peer reviewed journal papers, generated >£10 M of research income in the last 10 years and holds 2 patents on MFC stack development, configuration, modulation and control*.